# Extensive DNA End Processing by Exo1 and Sgs1 Inhibits Break-Induced Replication

**DOI:** 10.1371/journal.pgen.1001007

**Published:** 2010-07-08

**Authors:** Vanessa A. Marrero, Lorraine S. Symington

**Affiliations:** 1 Department of Genetics and Development, Columbia University Medical Center, New York, New York, United States of America; 2 Department of Microbiology and Immunology, Columbia University Medical Center New York, New York, United States of America; University of Washington, United States of America

## Abstract

Homology-dependent repair of DNA double-strand breaks (DSBs) by gene conversion involves short tracts of DNA synthesis and limited loss of heterozygosity (LOH). For DSBs that present only one end, repair occurs by invasion into a homologous sequence followed by replication to the end of the chromosome resulting in extensive LOH, a process called break-induced replication (BIR). We developed a BIR assay in *Saccharomyces cerevisiae* consisting of a plasmid with a telomere seeding sequence separated from sequence homologous to chromosome III by an I-SceI endonuclease recognition site. Following cleavage of the plasmid by I-SceI *in vivo*, *de novo* telomere synthesis occurs at one end of the vector, and the other end invades at the homologous sequence on chromosome III and initiates replication to the end of the chromosome to generate a stable chromosome fragment (CF). BIR was infrequent in wild-type cells due to degradation of the linearized vector. However, in the *exo1Δ sgs1Δ* mutant, which is defective in the 5′-3′ resection of DSBs, the frequency of BIR was increased by 39-fold. Extension of the invading end of the plasmid was detected by physical analysis two hours after induction of the I-SceI endonuclease in the wild-type *exo1Δ, sgs1Δ*, and *exo1Δ sgs1Δ* mutants, but fully repaired products were only visible in the *exo1Δ sgs1Δ* mutant. The inhibitory effect of resection was less in a plasmid-chromosome gene conversion assay, compared to BIR, and products were detected by physical assay in the wild-type strain. The rare chromosome rearrangements due to BIR template switching at repeated sequences were increased in the *exo1Δ sgs1Δ* mutant, suggesting that reduced resection can decrease the fidelity of homologous recombination.

## Author Summary

DNA double-strand breaks (DSBs) can occur spontaneously in cells by defective DNA replication or are induced by various types of DNA damaging agents, such as those used in chemo- or radiation therapy. Failure to repair DSBs, or inappropriate repair, can result in chromosome loss or chromosome rearrangements, events associated with development of cancer cells. Typically, DNA DSBs have two ends that are reunited faithfully by copying from a homologous donor chromosome. For DSBs that present only one end, repair occurs by invasion into a homologous sequence followed by replication to the end of the chromosome, a process called break-induced replication (BIR). This repair pathway is thought to be suppressed at two-ended DSBs to prevent extensive loss of heterozygosity. Here, we describe a new assay to physically monitor BIR to see how this repair pathway differs from the repair of two-ended DSBs. We show this pathway is infrequent, but can be detected by eliminating factors (Exo1 and Sgs1) that degrade linear DNA. However, increased chromosome rearrangements were found in the resection-defective strain. We found the kinetics of strand invasion in two-ended and one-ended DSB repair were the same, suggesting that the distinction between these pathways occurs after initial repair steps have begun.

## Introduction

DNA double-strand breaks (DSBs) are highly cytotoxic lesions that arise spontaneously during cell growth or following exposure to DNA damaging agents, such as ionizing radiation (IR). The repair of DSBs is critical for cell survival and maintenance of genome integrity. There are two major pathways to repair DSBs: non-homologous end joining (NHEJ) and homologous recombination (HR) [1]. NHEJ involves the religation of the two ends of the broken chromosome, which can occur with high fidelity or be accompanied by gain or loss of nucleotides at the junction [2]. HR relies on the presence of a homologous duplex to template repair of the broken chromosome and is generally considered to be error-free. However, HR can lead to a local loss of heterozygosity (LOH) if the homologous sequences are not identical, and to extensive LOH if repair is associated with a crossover. Furthermore, if repeats are utilized as the sequence donor and recombination is associated with crossing over, translocations can occur [3], [4]. If both ends of the DSB share homology with the donor duplex sequence, HR proceeds by a two-ended mechanism, such as double strand break repair (DSBR) or synthesis dependent strand annealing (SDSA) [1]. However, if coordination of the two ends is not maintained or only one end of the break is available, such as at a critically short telomere, repair can occur by break-induced replication (BIR) [5], [6]. In this case, following strand invasion replication occurs to the end of the chromosome to generate a stable repaired product. This can cause very long gene conversion tracts and significant LOH, and non-reciprocal translocations if invasion occurs at a dispersed repeated sequence [5], [7]–[10].

The repair of DSBs by HR requires the 5′-3′ nucleolytic degradation of the DNA ends to form invasive 3′ single-stranded DNA (ssDNA) tails. This processing reaction occurs in two steps: the first is catalyzed by the Mre11-Rad50-Xrs2 (MRX) complex and Sae2, which function together to clip sequences from the 5′ ends in increments of around 20–100 nt, the second step involves processive resection of the 5′ ends by Exo1 or Sgs1-Dna2 to form long tracts of ssDNA [11]–[14]. In the absence of Exo1 and Sgs1, or Exo1 and Dna2, partially processed intermediates with 3′ ssDNA tails of about 100–700 nts accumulate [12], [14]. The 3′ ssDNA tails created by end resection are bound by Rad51 to form a nucleoprotein filament that searches for homology and promotes pairing between the ssDNA bound by Rad51 and complementary sequences in the donor duplex [1]. The invading 3′ end is then used to prime DNA synthesis templated by the donor sequence. To complete two-ended repair two short tracts of leading strand DNA synthesis are required, whereas BIR requires extensive leading and lagging strand DNA synthesis [15]–[18].

Telomeres represent a natural source of one-ended DSBs. These are normally protected from recombination by telomere capping proteins, but when telomeres become critically short, for example, in the absence of telomerase, telomere maintenance depends on homologous recombination and this is thought to occur by BIR [6]. The analysis of telomere maintenance in the absence of telomerase revealed the importance of the *RAD52* epistasis group genes and *POL32* for BIR at telomeres [17], [19]–[22].

Because two-ended DSBs are repaired primarily by gene conversion, specialized assays have been developed to study BIR at non-telomeric sites in which only one end of a DSB can undergo homology dependent strand invasion. In one assay system, repair of an HO endonuclease induced DSB at the *MAT* locus was forced to occur by BIR from the chromosome homologue because homologous sequences on one side of the DSB had been deleted in diploid cells, or haploid cells disomic for chromosome III [16], [23]. Strand invasion and DNA synthesis resulted in copying sequences from the donor chromosome to the telomere. Using this system it was shown that strand invasion and completed repair, monitored by PCR and Southern blot analysis, respectively, is slower than repair by gene conversion. Haploid assay systems have been developed using the HO cut site inserted at an ectopic site so that non-essential sequences centromere distal to the cut site can be lost without compromising viability, and strand invasion using homologous sequences on only one side of the DSB can restore a telomere by BIR [17], [24], [25]. In these assays, the kinetics of repair as determined by extension of the invading strand were slow, even though Rad51 loading and pairing with the donor sequence were rapid, suggesting cells sense whether DSBs have a second end to complete repair by gene conversion and delay initiation of DNA synthesis when only one end is present [25].

Another approach to study BIR involves transforming yeast cells with a linearized plasmid that contains a telomere seeding sequence on one end and a region of homology to unique sequences present on a yeast chromosome at the other end [26]. One end of the plasmid undergoes *de novo* telomere formation and the other is used for strand invasion and replication to the end of the chromosome. In this assay system, the strand invasion intermediate was shown to be unstable, as predicted for the SDSA model of recombination, and to undergo repeated cycles of strand invasion and dissociation before establishing a processing replication fork to complete replication of sequences downstream of the site of strand invasion [9]. These results suggest strand invasion during BIR is similar to gene conversion and extensive replication (BIR) occurs only after failure to capture the second end and repair by gene conversion. Template switching during BIR has also been shown to occur during repair of a chromosomal DSB resulting in triparental translocations [27].

These two hypotheses for how BIR initiates differ considerably in that one predicts there is a delay in the initiation of DNA synthesis from the strand invasion intermediate when the other end of the break to engage in repair is absent, the other predicts extension of the invading strand is independent of the other end and is terminated by dissociation of the extended invading strand pairing with the other side of the DSB. If there were no end to pair with then strand invasion would occur again and eventually lead to formation of a processive replication intermediate.

The transformation assay has several limitations: it cannot be used to study the kinetics of repair, or to identify repair intermediates, because less than one percent of cells are competent for transformation and the synchrony of DNA uptake is not known. In order to overcome these problems, we generated a new assay where the plasmid is cut *in vivo* by the I-SceI endonuclease. BIR occurs with low frequency in this system because the Exo1 and Sgs1-dependent resection pathways degrade the linearized plasmid. Inhibition of extensive end resection using the *exo1Δ sgs1Δ* double mutant enabled BIR products to be detected by physical assays. The inhibitory effect of resection was less in a plasmid-chromosome gene conversion assay, compared to BIR, and products were detected by physical assay in the wild type strain. By comparing the kinetics of strand invasion and extension of the invading strand in the plasmid BIR and gene conversion assays we observed no significant difference between BIR and gene conversion repair suggesting the initiation of both processes is similar.

## Results

### Plasmid-based BIR assay

We previously used a plasmid transformation assay to study the mechanism and genetic control of BIR [9], [26], [28]. The chromosome fragmentation vector (CFV1) contains the *URA3* selectable marker, *SUP11*, *CEN4*, an *ARS*, a tract of (G_1–3_T)_n_ to provide a site for telomere addition and a unique DNA segment from the left arm of chromosome III (*BUD3*). The CFV is linearized between *BUD3* and the telomere seeding sequences with a restriction endonuclease and used to transform yeast, selecting for Ura^+^ colonies. Most transformants (95%) arise by *de novo* telomere addition to heal one end of the CFV and strand invasion at the other end into the endogenous yeast locus to copy the entire chromosome arm yielding a stable 110-kb chromosome fragment (CF). This BIR assay involves transformation of chemically competent yeast cells with a linearized CFV and selection for completed repair events. This system is not useful for physical analysis of intermediates and products of BIR because only 0.1% of cells are competent for transformation, the synchrony of DNA uptake is not known and multiple copies of the linearized vector could enter the cell. Furthermore, because naked DNA is used as a substrate it is possible that the efficiency and mechanism of repair could be different to a chromatinized template.

**Figure 1:**
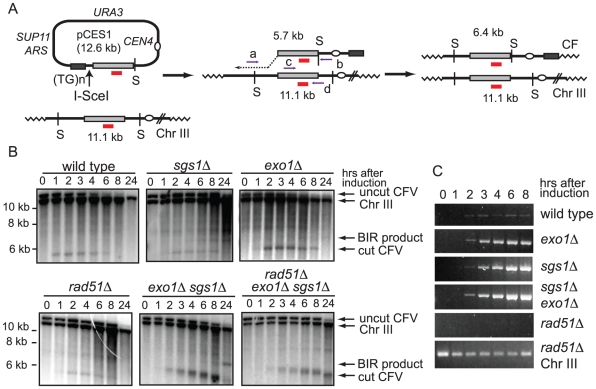
Break-induced replication assay. (A) Schematic of the assay: I-SceI induced cleavage of pCES1 (large arrow) generates a linear fragment with one end containing a telomere seeding sequence ((TG_1-3_)_n_) and the other is homologous to the *BUD3* region of chromosome III. Strand invasion and DNA synthesis (dotted arrow) extends the pCES1 to form the completed repair product designated as the Chromosome Fragment (CF). S indicates the location of *Spe*I cut sites and the sizes of the DNA fragments after *Spe*I digestion are shown. The chromosome III probe is designated by a short red line; locations for PCR primers are shown by horizontal arrows; zigzag lines indicate telomeric sequences. (B) Southern blots showing cleavage of the vector and formation of the novel *Spe*I fragment indicative of BIR at different times following induction of I-SceI. Representative gels from multiple trials are shown. (C) PCR (primers a and b) to detect the primer extension product during BIR. The bottom panel shows the control PCR using primers c and d amplifying part of the *BUD3* locus to show the presence of DNA in the *rad51Δ* samples.

To circumvent these limitations the CFV was modified by introduction of the I-SceI endonuclease recognition site between the telomere seeding sequences and the sequences with homology to the *BUD3* region of chromosome III (Figure 1A). I-SceI was used instead of HO to overcome the requirement of using yeast strains with the native HO cut sites deleted. This plasmid (pCES1) was used to transform a yeast strain containing an integrated *P_GAL_*:*I-SCEI* cassette. The plasmid was maintained as an episome until induction of the I-SceI endonuclease. Cleavage of the plasmid and subsequent repair were detected by Southern blot analysis of *Spe*I-digested genomic DNA isolated from samples taken prior to I-SceI induction and at 1–2 hr intervals after addition of 2% galactose to the culture. pCES1 has a unique *Spe*I site resulting in a 12.6 kb fragment prior to I-SceI cleavage; of the two fragments produced by I-SceI digestion only the 5.7 kb fragment hybridizes to the *BUD3* probe. Initiation of repair by BIR should result in a novel 6.4 kb *Spe*I fragment (Figure 1B). Linearization of the vector by I-SceI was detected at the 1 hr time point and the 5.7 kb cut fragment was visible until 6 hr (Figure 1B). However, we were unable to detect formation of the 6.4-kb SpeI fragment indicative of BIR. By 8 hr the cut fragment had disappeared and the band corresponding to uncut plasmid was very faint indicating most of the plasmids in the population had been cut and degraded. Plasmid loss following I-SceI induction was confirmed by PCR using primers specific for the vector (data not shown).

To determine whether BIR can occur at low frequency cells were plated after I-SceI induction on either synthetic complete (SC) medium or SC-URA medium to detect retention of the plasmid. Ura^+^ colonies from each time point were then tested for mitotic stability to distinguish between stable CFs formed by BIR and uncut or end-joined plasmids [26]. Although maintained under selection, only 30–50% of the cells were Ura^+^ before I-SceI induction. The plasmid was rapidly lost after induction of I-SceI with only 1% of the population remaining Ura^+^ 24 hr after I-SceI induction, and of these colonies about 10% were due to CF formation (verified by pulsed field gel electrophoresis (PFGE)).

### 5′-3′ resection by Exo1 and Sgs1 is inhibitory to BIR

Malkova et al [23] have shown that BIR is a slow repair process; thus, it is possible that the 12.6 kb linearized plasmid is completely degraded before BIR initiates. To determine whether degradation of the substrate was the cause for low BIR frequency we tested the *exo1Δ, sgs1Δ* and *exo1Δ sgs1Δ* mutants in the BIR assay. These mutants were chosen because resection is initiated by MRX-Sae2 forming a short 3′ ssDNA tailed intermediate that is a substrate for Rad51-catalyzed strand invasion, but long range resection is prevented in the *exo1Δ sgs1Δ* double mutant [12], [14]. While improvements in plasmid retention and the BIR frequency as determined by plating efficiency were observed in the single mutants, the double mutant showed the largest increase with 7.0% of the resulting colonies containing CFs, compared with 0.18% for wild type (Table 1). Furthermore, a faint band corresponding to the extension product was detected by Southern blot at 8 hr after I-SceI induction in the *exo1Δ sgs1Δ* double mutant (Figure 1B). Only one CF was detected among 171 rare Ura^+^ colonies formed 24 hr after I-SceI induction in the *sgs1Δ exo1Δ rad51Δ* mutant indicating that the increase in BIR was dependent on *RAD51*. To ensure that the Exo1 nuclease activity was responsible for the reduction in BIR, the *exo1Δ* mutant was transformed with plasmids expressing either wild type *EXO1* or the *exo1-D173A* allele [29]. Only the strain expressing the nuclease-defective *exo1-D173A* allele showed elevated BIR confirming that the Exo1 nuclease is responsible for low frequency BIR (Table 1).

**Table 1:** *EXO1* and *SGS1* inhibit BIR.

Relevant genotype	Percent BIR^a^±SEM	Relative frequency^b^
*EXO1 SGS1*	0.18±0.04	1
*exo1Δ*	0.74±0.14*	4
*sgs1Δ*	0.40±0.08	2
*exo1Δ sgs1Δ*	7.03±2.69*	39
*rad51Δ*	<0.01*	—
*exo1Δ sgs1Δ rad51Δ^C^*	0.006*	0.03
*exo1Δ + pEXO1*	0.23±0.07	1
*exo1Δ + pexo1D173A*	1.23±0.35*	7
*sgs1-D664Δ*	0.22±0.12	1
*exo1Δ sgs1-D664Δ*	12.21±1.25*	68

^**a**^The percent BIR was determined by the number of stable Ura^+^ colonies divided by the number of Ura^+^ colonies at 24 hr normalized to the percent Ura^+^ at 0 hr.

^**b**^All fold differences are calculated with respect to wild type.

^**c**^One CF was recovered from 171 Ura^+^ colonies scored from three independent trials.

*Designates values which show statistically significant difference from wild type, *P*-values <0.05.

In a PCR assay to measure primer extension during BIR the product was barely detectable in the wild type strain and did not increase over time, but was readily detected in the resection defective *exo1Δ, sgs1Δ* and *exo1Δ sgs1Δ* strains (Figure 1C). A *rad51Δ* mutant was used as a control and no PCR product was detected in this strain. The PCR product diagnostic of extension of the invading 3′ end was detected 2 hr after I-SceI induction in the *exo1Δ sgs1Δ* double mutant, earlier than BIR was detected by Southern blot analysis. The PCR assay can detect extension of the 3′ end of the strand invasion intermediate as well as completed products, whereas the Southern blot assay requires the invading end become dsDNA to be cleaved by *Spe*I; therefore the delayed product formation in the Southern blot assay could be due to a delay in initiating lagging strand synthesis. However, it is more likely that the apparent difference in timing is due to the higher sensitivity of PCR compared with the Southern blot assay.

**Figure 2:**
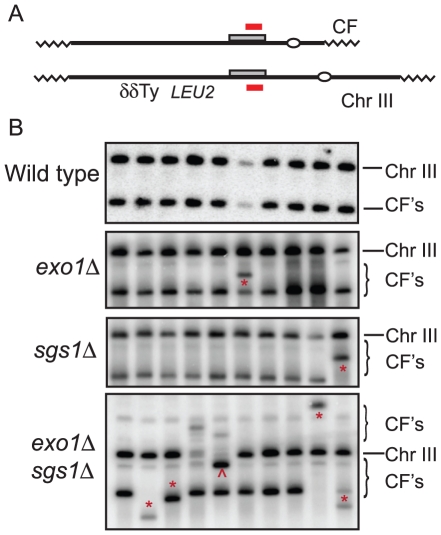
PFGE and Southern blot analysis of individual CFs. (A) Schematic of completed CF and Chr. III showing the location of the Ty and δ elements downstream of the site of strand invasion by the CFV. The shaded rectangle shows the region of homology shared by the vector and chromosome III, the short solid line designates the hybridization probe. (B) Stable Ura^+^ colonies from the BIR assay were analyzed by PFGE and Southern blot hybridization. * Indicates unexpected size CFs, ∧ indicates unusual size chromosome III.

Analysis of 34 CFs recovered from the wild type strain by PFGE and Southern blot analysis revealed that all were of the expected size of 110 kb (Figure 2); in two of the samples, the mobility of chromosome III was altered suggesting strand invasion can result in chromosome III rearrangements. These have not been analyzed further. Because chromosome III rearrangements are rarely seen in the wild-type strain, and the two events recovered were of the same size and from the same induction, it is possible that these are clonal events. Two of 30 independent repair events analyzed for the *exo1Δ* mutant exhibited aberrant-sized CFs. From the *sgs1Δ* mutant, three clones had aberrant-sized CFs and two had chromosome III rearrangements (Figure 2). Twelve CFs analyzed from the *exo1Δ sgs1Δ* double mutant were of aberrant sizes and one clone had a chromosome III rearrangement (13 out of 30 total aberrant events), a significant increase compared with wild type (*P* = 0.0007) and the single mutants (*P*<0.05). The CFs of different sizes are likely to result from elevated template switching at the cluster of Ty/δ elements near the *LEU2* locus [9]. Some of the Ura^+^ colonies had two different sized CFs; these could be due to two independent repair events in cells with two copies of the CFV or be formed by secondary recombination events. For samples with one prominent CF band and a fainter CF hybridizing signal we assume the latter is due to a secondary event and these are not marked as aberrant events.

The negative effect of resection on BIR was also seen in the transformation-based BIR assay. In this system the frequency of BIR is determined by the number of Ura^+^ colonies derived from transformation of cells with the linear CFV compared to an uncut replicating plasmid to account for transformation efficiency [26]. A derivative of the CFV lacking the *ARS* element (pLS192) was used to eliminate the background of Ura^+^ colonies due to NHEJ. The *exo1Δ* and *exo1Δ sgs1Δ* mutants both exhibited a significant increase in the BIR frequency compared with wild type (Table 2).

**Table 2:** Frequency of BIR in the transformation assay.

Relevant genotype	Frequency of BIR^a^ ± SEM	Relative frequency^b^
*EXO1 SGS1*	0.6±0.16	1
*exo1Δ*	10.2±3.7*	17
*sgs1Δ*	5.2±1.8	9
*exo1Δ sgs1Δ*	25.0±4.4*	42
*rad51Δ*	0.06±0.02*	0.1
*sgs1-D664Δ*	1.7±0.7	3
*exo1Δ sgs1-D664Δ*	21.2±2.5*	35

^**a**^The frequency of BIR is the number of Ura^+^ transformants per microgram of linearized pLS192 DNA transformed divided by the number of Ura^+^ transformants per microgram of circular pRS416 transformed.

^**b**^All fold differences are calculated with respect to wild type.

*Designates values which show statistically significant difference from wild type, *P*-values <0.05

### The *sgs1-D664Δ* mutation improves the efficiency BIR

**Figure 3:**
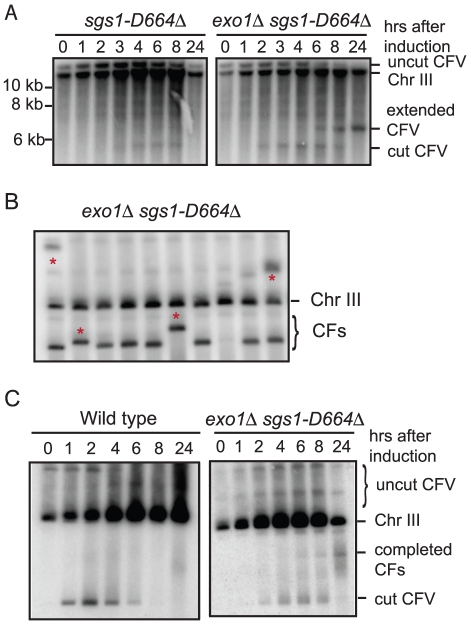
Physical analysis of BIR in the *exo1Δ sgs1D664Δ* double mutant. (A) Southern blot analysis as in Figure 1 for the *sgs1-D664Δ* and *exo1Δ sgs1D664Δ* mutants. (B) PFGE and Southern blot analysis of stable Ura^+^ colonies derived from pCES1. (C) PFGE analysis of CF formation following I-SceI induction.

One problem with using the *exo1Δ sgs1Δ* double mutant for further physical analysis of BIR is the increased number of aberrant CFs generated; in addition, the strain exhibits slow growth. Bernstein et al [30] described an *sgs1* separation of function allele, *sgs1-D664Δ*, which suppresses the *top3Δ* slow growth defect, but does not exhibit spontaneous hyper-recombination or DNA damage sensitivity. We have analyzed DSB processing in the *exo1Δ sgs1-D664Δ* double mutant and found there is a defect in the processivity of resection resulting in ssDNA tails of around 5-kb (E. Mimitou and K. Bernstein, unpublished data). To determine whether this might provide the benefits of enhanced BIR by preventing complete degradation of the linear CFV, but without the hyper-recombination phenotype, we analyzed the kinetics and frequency of BIR in the *exo1Δ sgs1-D664Δ* double mutant. By Southern blot analysis and in the plating assay the frequency of BIR was similar to the *exo1Δ sgs1Δ* mutant (Figure 3A and Table 1). The 6.4 kb *Spe*I fragment, corresponding to the extended CFV, was clearly detected 6 hr after I-SceI induction and 4 hr after the appearance of the I-SceI cut fragment. The faster appearance of the BIR product compared with the *exo1Δ sgs1Δ* mutant could be because there is more resection creating a better substrate for Rad51-catalyzed strand invasion. High frequency BIR, comparable to the *exo1Δ sgs1Δ* mutant, was also observed in the transformation assay (Table 2). Analysis of CFs recovered from the *exo1Δ sgs1-D664Δ* double mutant revealed 6 aberrant-sized CFs of 29 events analyzed (Figure 3B). The number of total aberrant events recovered from the *exo1Δ sgs1-D664Δ* strain is not significantly different to the wild-type strain (*P* = 0.13) or the *exo1Δ sgs1Δ* mutant (*P* = 0.09), but if only the aberrant-sized CFs are considered then the difference between wild type and *exo1Δ sgs1-D664Δ* is significant (*P* = 0.007).

Because the *exo1Δ sgs1-D664Δ* mutant exhibits faster growth than the *exo1Δ sgs1Δ* mutant, but still shows increased frequency BIR, we tested whether completed BIR repair events were formed during the time course of I-SceI induction by PFGE. A faint band corresponding to the 110 kb CF was detected as early as 6 hr after I-SceI induction in the *exo1Δ sgs1-D664Δ* strain, but, as expected, was not seen in wild type. This result shows that the appearance of the 6.4 kb SpeI fragment by Southern blot hybridization correlates with the formation of completed repair products.

### Telomere addition is more efficient in the *exo1Δ sgs1Δ* double mutant

**Figure 4:**
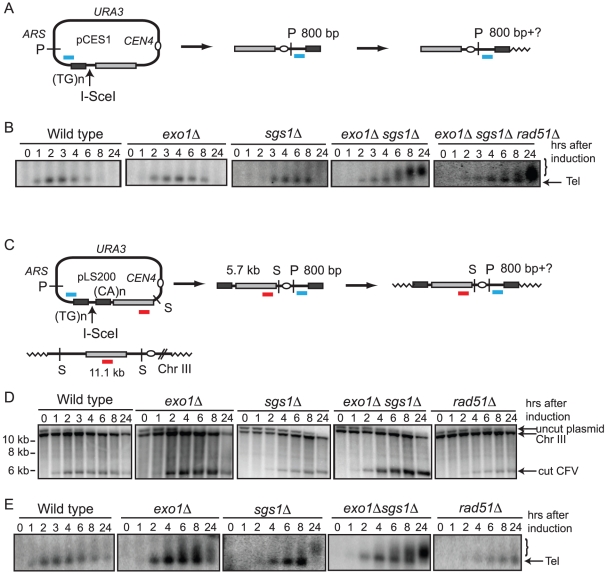
Analysis of telomere addition. (A) Schematic of telomere addition to pCES1 following I-SceI cleavage. The probe for the terminal fragment is designated by a short blue line, P indicates *PstI* restriction sites, telomeres are indicated with zigzag lines. (B) Southern blot analysis of telomere addition to the terminal PstI fragment containing the telomere seeding sequence. The bracket indicates the heterogeneous band due to addition of telomere repeats to the seed sequence. (C) Schematic of the double-telomere vector and assay. The plasmid is linearized by I-SceI to form a linear fragment with telomere seeding sequence at each end. The probe to detect linearization of the vector is shown in red and the probe for the telomere fragment on the right indicated by the blue line. S and P indicate restriction endonuclease sites for *Spe*I and *Pst*I, respectively. (D) Southern blots of *Spe*I-digested genomic DNA hybridized with the Chr. III (red) probe. (E) Southern blots of *Pst*I-digested DNA to identify addition of telomere repeats to the right telomere seed sequence. Arrow indicates the *Pst*I fragment containing the telomere seeding sequence, the bracket indicates the heterogeneous band due to addition of telomere repeats to the seed sequence.

CF formation requires strand invasion at one end of the vector and *de novo* telomere addition at the other end. Thus, the low frequency of CF formation could be due to a defect in one or both of these processes. To analyze telomere addition, DNA isolated from cells after I-SceI induction was digested with *Pst*I to liberate the terminal fragment containing the telomere seeding sequence (Figure 4A). *De novo* telomere addition to this end of the linearized CFV should result in a shift to a higher molecular weight form [31]. Because telomere length is variable we expected to observe a heterogeneous population of fragments up to 300 bp longer than the original 800 bp fragment. We were not able to detect extended products resulting from telomere addition at the seeding site in wild type, *exo1Δ* or *sgs1Δ* strains. However, in the *exo1Δ sgs1Δ* double mutant telomere addition was detected 6–8 hr after linearization of the plasmid (Figure 4B). This raised the concern that inefficient telomere recognition and/or elongation might contribute to the low frequency CF formation in the wild-type strain. To test this, a plasmid with telomere seeding sequences on either side of the I-SceI site was constructed (Figure 4C). When this plasmid was linearized *in vivo* in the wild type, *exo1Δ, sgs1Δ,* and *exo1Δ sgs1Δ* strains the linear form was still detected at the 24 hr time point indicating the ends are recognized as telomeres and protected from degradation. However, it was still difficult to see addition at the telomere-seeding site in the wild type and the *exo1*Δ strains (Figure 4D). The improved telomere addition in the *exo1Δ sgs1Δ* mutant is most likely due to the reduced resection providing a short ssDNA tail that can be recognized by Cdc13 [31]. The end protection was not due to homologous recombination because it was also seen in the *rad51*Δ strain, which is defective for inter-chromosomal homologous recombination [26], [32].

### The *exo1Δ* mutation improves the efficiency of plasmid gene conversion

**Figure 5:**
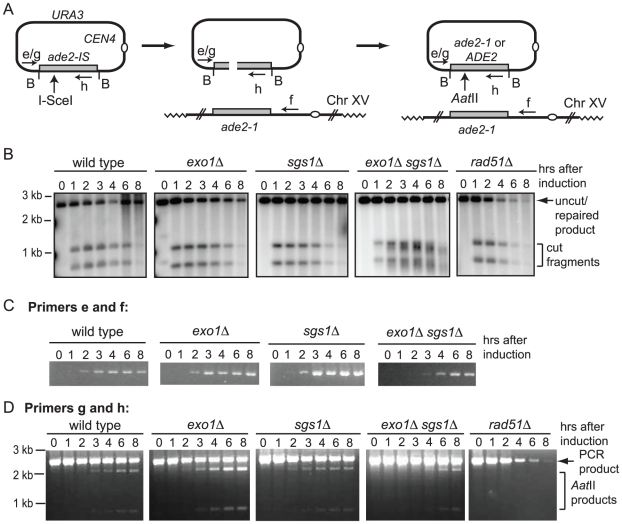
Gene conversion assay. (A) Schematic of the gene conversion assay. I-SceI cleaves the plasmid to generate a linear fragment with homology to the *ADE2* locus at both ends of the break. Repair from the genomic locus results in restoration of the *Aat*II site, replacing the I-SceI site. The location of PCR primers used to identify extension of the invading strand strand and completed products are shown by arrows labeled e, f, g and h. (B) Southern blot of genomic DNA digested with *Bam*HI (B) to liberate a 3.7 kb fragment containing the plasmid *ade2* allele. Cleavage by I-SceI results in fragments of 2.0 and 1.7 kb and repair restores the 3.7 kb fragment. (C) PCR to detect initiation of DNA synthesis from the left invading end at different times after induction of I-SceI. (D) PCR with primers g and primer h to amplify the plasmid *ade2* allele followed by *Aat*II digestion to detect repair of the I-SceI site.

To determine whether degradation of short linear substrates is a general problem in ectopic recombination assays, or specific to BIR, we created a plasmid substrate with an I-SceI cut site within the *ADE2* gene to monitor repair by gene conversion from a chromosomal donor sequence (*ade2-1*) (Figure 5A). Following cleavage of the plasmid-borne *ade2-IS* allele, repair from the chromosomal allele restores the *Aat*II site that was disrupted by the I-SceI cut site insertion. Cells from each plasmid-containing strain were plated onto SC and SC-URA media at different time points after induction of the I-SceI endonuclease. The number of Ura^+^ colonies formed 8 hr after induction of I-SceI was reduced by ten-fold in the wild-type strain, by five-fold in the *sgs1Δ* mutant, but only two to three-fold in the *exo1Δ* and *exo1Δ sgs1Δ* mutants. Only 4% of the *rad51Δ* cells retained the plasmid 8 hr after I-SceI induction indicating that plasmid loss is due to inefficient repair. Southern blot analysis of *Bam*HI digested genomic DNA confirmed that I-SceI cleavage products are generated in all of the strains (Figure 5B). There was delay in I-SceI cutting in the *exo1Δ sgs1Δ* mutant and the characteristic smearing of the bands due to partial resection was observed [12], [14]. Repair of the plasmid DSB results in restoration of the *Bam*HI fragment, and this band was present 8 hr after I-SceI induction in all the strains except the *rad51Δ* mutant.

To determine the kinetics of strand invasion, primers were designed to anneal to the plasmid upstream of the homology region and to the genomic locus downstream of the homology region. These primers should amplify the product formed by strand invasion and DNA synthesis beyond the region of homology shared by the plasmid and chromosome XV, and also completed events that result in plasmid integration. Studies of plasmid gap repair indicate that 20–50% of the repair events are associated with integration of the plasmid [33]–[35]. Although plasmid integration would lead to formation of an unstable dicentric chromosome, these events are likely to persist through the 8 hr time course. For the wild type and single mutants, the PCR product was detected 2 hr after induction of I-SceI, and at 3 hr in the *exo1Δ sgs1Δ* double mutant (Figure 5C). No PCR product was seen in a *rad51Δ* strain (data not shown).

Completed repair products can also be detected by PCR using primers that anneal to plasmid sequences upstream of the homology region and within *ade2*, downstream of the I-SceI cut site. These primers amplify the uncut plasmid allele, invasion intermediates and completed gene conversion products, but only intermediates and completed repaired product will be digested with *Aat*II (Figure 5D). In contrast to BIR, gene conversion products were detected by the physical assay in the wild type strain, appearing 3 hr after inducing I-SceI, similar to the repair kinetics reported for other ectopic recombination assays [36]. Similar results were obtained using primers that anneal to plasmid sequences flanking the *ade2* insert that are specific for uncut plasmid or completed repair events (data not shown). Repaired products were detected with similar kinetics in the *exo1Δ* and *sgs1Δ* mutants, but were delayed in the *exo1Δ sgs1Δ* double mutant. The delay in detection of strand invasion intermediates and completed products in the *exo1Δ sgs1Δ* mutant may be due to the delay in I-SceI cutting and reduced resection. I-SceI cutting is complete at 8 hr in the *exo1Δ* mutant and plasmid retention is around 30%, compared with 10% for the wild type strain; thus, the frequency of gene conversion is increased by three-fold in the absence of Exo1. The increased cleavage of the PCR product by *Aat*II in the *exo1Δ* mutant is also diagnostic of increased repair compared with wild type and the *sgs1Δ* mutant (Figure 5D).

## Discussion

We have developed a plasmid-based assay system to study BIR as an alternative to chromosomal systems. This assay utilizes features of the previously described plasmid transformation system except the initiating DSB is made *in vivo* instead of by transforming linearized plasmid DNA into cells. The goal of developing this system was to physically monitor BIR using an assay that could be easily moved into different strain backgrounds; however, we discovered that a major limitation to the assay was degradation of the linearized substrate. Although this could be overcome by eliminating the Exo1 nuclease and Sgs1 helicase, which control degradation of linear DNA [11], [12], [14], the absence of Exo1 and Sgs1 resulted in elevated chromosome rearrangements during BIR. The inhibitory effect of DNA end resection by Exo1 was also observed using a plasmid-chromosome gene conversion assay.

In this study we found a much lower frequency of BIR in the wild type strain (<1%) than observed in chromosomal ectopic BIR assays (10–30%) [17], [25]. The low frequency may be due to inefficient cleavage of the plasmid by I-SceI, the low copy number of the plasmid, and the requirement for telomere addition at the telomere seed sequence at one end of the linearized vector. Although a plasmid with two telomere seeding sequences flanking the DSB was maintained as a linear plasmid following linearization it was not 100% efficient (Figure 4). During the course of these studies we found that the *SUP11* gene present on the vector is responsible for the low plasmid retention. Thus, it is possible that the system could be improved by using the HO cut site instead of I-SceI and by replacing the short telomere seed sequence (43 bp) with a longer one in a vector lacking *SUP11*.

Deleting the *EXO1* and *SGS1* genes resulted in a significant increase in the frequency of BIR, compared with the wild type strain (Table 1 and Table 2). We attribute this effect to the role of both proteins in regulating the resection of DSBs because the linearized plasmid was still present 8 hr after I-SceI cleavage in the *exo1Δ sgs1Δ* double mutant, but was undetectable in the wild-type strain (Figure 1). Furthermore, the Exo1 nuclease activity was responsible for reduced BIR and the resection-defective *sgs1-D664Δ exo1Δ* mutant behaved the same as the *sgs1Δ exo1Δ* mutant. The *sgs1*Δ mutation conferred less of a suppressive effect than the *exo1Δ* mutation, but together the two mutations synergized to increase the frequency of BIR. In the absence of Exo1 and Sgs1 partial resection of DSB ends occurs to form 3′ single-stranded DNA (ssDNA) tails of around 100–700 nucleotides [12], [14]. These intermediates can be used for gene conversion repair albeit with slight reduced efficiency compared with resection proficient strains [12], [14]. Thus the increased efficiency of BIR in the *exo1Δ sgs1Δ* double mutant appears to be due to sufficient resection to form an invasive ssDNA end, but without the extensive resection that completely degrades the plasmid. Although the resection defect of the *exo1Δ sgs1-D664Δ* mutant is not as severe as the *exo1Δ sgs1Δ* mutant (E. Mimitou and K. Bernstein, unpublished results), the more extensive resection appears to create a better substrate for Rad51-catalyzed strand invasion and the linearized plasmid is stable for long enough to allow BIR to occur. Preventing extensive resection was also found to increase the retention of a linearized plasmid that can repair by gene conversion. The increased stability of short linear DNA fragments in the *exo1Δ sgs1Δ* double mutant could also explain the increase in plasmid integration during transformation reported previously, and reduced resection might be more favorable for spontaneous recombination between short repeats [37], [38].

Extensive resection would be expected to be less problematic in the chromosomal assay systems compared with the plasmid assay because of the much larger substrates used. However, even when generating a DSB on chromosome III with extensive homology available on one side of the DSB to initiate BIR, more than 50% of the repair events occur by resection of about 30-kb from the HO cut site to a pair of inverted Ty elements proximal to the *MAT* locus (FS2) [10], [23]. The ssDNA formed at these elements undergoes intramolecular annealing, or intermolecular annealing between sister-chromatids, to form inverted dimers that undergo complex rearrangements before yielding viable products. In the ectopic assays involving short homologies, extensive resection could expose repeated sequences, such as δ and Ty elements, that could be used to initiate recombination with repeats elsewhere in the genome. Furthermore, the 3′ ends of resected intermediates are unstable several hours after induction of HO endonuclease [39]; thus, the small region of homology required for strand invasion could be lost resulting in lower frequency BIR. Indeed, elimination of Exo1 or Sgs1 also increases the efficiency of BIR in an ectopic chromosomal assay [40].

The *sgs1* mutation has been shown to increase the percent of DSBR events that resolve as crossovers [41]. Thus, one possible explanation for the increase in events that result in CFs in the *exo1Δ sgs1Δ* double mutant would be increased resolution of the strand invasion intermediate to form a crossover linking the CFV to the left arm of chromosome III. We have previously shown that mutations in the Polymerase δ complex result in CFs from half-crossovers instead of BIR [18]. However, when a diploid *exo1Δ sgs1Δ* strain was used in the BIR assay we did not find increased loss of one copy of chromosome III as predicted from half crossovers (data not shown). Furthermore, a simple crossover between the CFV and left arm of chromosome III would generate CFs of uniform size, contrary to our findings (Figure 2). The other possible explanation for increased stability of the linear CFV would be *de novo* telomere addition at the I-SceI cut site, as shown to occur at an HO-induced DSB in *exo1Δ sgs1Δ* mutants [40], [42]. Short linear plasmids do not exhibit high mitotic stability in *S. cerevisiae* so these events would not have been scored as stable Ura^+^ colonies [43]. In addition, the physical analysis of CFs in the *exo1Δ sgs1Δ* and *exo1Δ sgs1-D664Δ* mutants showed most of the aberrant-sized CFs were larger than the expected size of 110-kb, instead of the 13.2-kb product expected from telomere addition at both ends of the linear vector. Furthermore, because there is no replication origin present on the vector used in the BIR transformation assay all the transformants must arise by strand invasion to copy or link a chromosomal origin to the vector and cannot arise by telomere addition at both ends of the vector. Thus, *de novo* telomere addition at the I-SceI site appears to be infrequent, and the large increase in events in the *in vivo* cutting and transformation assays in the *exo1Δ sgs1Δ* double mutant is consistent with stabilization of the short linear DNA resulting in more time for strand invasion to occur.

In the wild-type strain all of the CFs recovered were of the expected size from strand invasion at the *BUD3* locus and replication to the end of chromosome III (Figure 2). Twenty to 40% of the CFs analyzed from the *exo1Δ sgs1-D664Δ* and *exo1Δ sgs1Δ* mutants, respectively, exhibited aberrant mobility by PFGE (Figure 2). Template switching at the Ty element or cluster of δ elements located 5-kb downstream of the region of homology is likely to be responsible for the novel CFs. If the end of the substrate is extruded as it passes through these repetitive elements, re-invasion might occur into any of the multiple copies of Ty or δ elements found scattered throughout the genome as reported previously using the plasmid transformation assay [9]. Consistent with this hypothesis, we did not recover unusual sized CFs from the *exo1Δ sgs1-D664Δ* mutant using a vector that invades a chromosome arm devoid of Ty and δ elements (data not shown).

Because we did not observe a significant increase in the number of aberrant CFs in the *sgs1Δ* single mutant, but the number was increased in the *exo1Δ sgs1Δ* and *exo1Δ sgs1-D664Δ* mutants, it appears to be due to the decrease in end resection instead of the increased crossovers [41]. However, another possibility is increased recombination between diverged sequences (homeologous) resulting in template switching to more diverged Ty/δ elements, a process suppressed by *SGS1* [44], [45]. In a study of gross chromosome rearrangements, combining the *sgs1Δ* mutation with defects in checkpoint functions or chromatin assembly factors resulted in complex rearrangements between the diverged *CAN1, LYP1* and *ALP1* loci, interpreted as BIR and multiple cycles of template switching between homeologous sequences [46]. The *exo1Δ* mutant also exhibits a slight increase in homeologous recombination [47], but the *exo1Δ sgs1Δ* double mutant has not been tested to determine whether the mutations synergize to allow more promiscuous recombination.

Previous studies showed a delay in extension of the 3′ end of the strand invasion intermediate in BIR compared with a two-ended repair event [23], [25]. For both processes the kinetics of Rad51 loading to the resected end and pairing with donor sequences were the same, suggesting the delay is at the step of DNA synthesis from the invading end [25]. In our assay we are unable to assess the kinetics of repair in the wild-type strain because the process is too inefficient to detect by Southern blot analysis. However, a weak product was detected two hours (2 hr) after I-SceI induction in the PCR primer extension assay (Figure 1). In the *exo1Δ sgs1Δ* mutant the PCR primer extension product was detected at 2 hr and the novel *Spe*I fragment indicative of repair detected at 8 hr. It is possible that the *Spe*I fragment is present earlier, but not detected due to the low sensitivity of the Southern blot assay. The kinetics of primer extension seen here are faster than reported in studies from the Haber lab [17], [23], [25]. In our assay only one end generated by the DSB is available for strand invasion and the telomere seeding sequence at the other end should be recognized by telomere binding proteins. Thus, one possible explanation for the more rapid detection of strand invasion intermediates in the plasmid assay is that the telomere end is not recognized as a broken chromosome and only one end is “seen” to be in need of repair.

In the gene conversion assay primer extension was detected at 2 hr in the wild type, *exo1Δ* and *sgs1Δ* strains, and at 3 hr in the *exo1Δ sgs1Δ* double mutant (Figure 5). The delay in the double mutant was likely due to less efficient cleavage of the plasmid by I-SceI and reduced resection to form the substrate for Rad51. Completion of repair was detected in the wild type and mutant strains at 3–4 hr, similar to other ectopic recombination assays [36]. The lower efficiency of BIR compared with gene conversion is consistent with failure at a step downstream of strand invasion [17], [25]. We suggest that following extension of the invading 3′ end by a short tract of DNA synthesis, the invading end dissociates and in the absence of a second end to anneal to, the linear plasmid is susceptible to more extensive degradation of the 5′ end before initiating a second strand invasion event [5]. This cycle could occur several times before assembly of the replisome and completion of BIR. Long 3′ ssDNA tailed intermediates are unstable [43]; thus, reducing the length of the 3′ tail by preventing resection of the 5′ strand may help to preserve the intermediate formed by dissociation resulting in more frequent BIR in the *exo1Δ sgs1Δ* double mutant. Similarly, stabilization of the linear intermediate following dissociation of the invading strand at repeated sequences could result in the higher frequency of aberrant-sized CFs in the resection-defective *exo1Δ sgs1Δ* double mutant (Figure 2). In the gene conversion assay only one round of strand invasion and extension of the 3′ end by DNA synthesis, followed by dissociation and annealing, would be necessary to form a stable repaired product.

## Materials and Methods

### Media, growth conditions, and genetic methods

Rich medium (yeast extract-peptone-dextrose (YPD)), synthetic complete medium (SC) lacking the appropriate amino acids or nucleic acid bases, sporulation medium, and genetic methods were as described previously [48]. Synthetic deficient medium (SD) containing 2% raffinose and supplemented with adenine, histidine, tryptophan, lysine, and leucine was used for the galactose induction of *I-SCEI*. Transformation of yeast was by the lithium acetate method [49]. Standard procedures were used for genetic crosses [48].

### Yeast strains and plasmids

*S. cerevisiae* strains used in this study were derived from W303 (*his3–11,15 leu2–3,112 trp1–1 ura3–1 ade2–1 can1–100)* and are listed in Table S1, only deviations from this genotype and the *MAT* allele are given [50]. The W303 derivative with the *lys2::P_GAL_-I-SCEI* cassette (Lev488) was a gift from S. Marcand. This strain was crossed to strains in the lab collection with *exo1::HIS3, sgs1::HphMX4, sgs1-D664D* or *rad51::LEU2* alleles to create haploid derivatives with the *lys2::P_GAL_-I-SCEI* cassette and mutations in the relevant genes.

Plasmid pCES1 was constructed by inserting the annealed oligonucleotides, CES1-2F and CES1-2R, carrying the I-SceI recognition sequence adjacent to a telomere seeding sequence, into *Bgl*II/*Hin*DIII digested pYCF/D8B plasmid [28] (Table S2). The annealed oligonucleotides BglII/Tel-ISceI/BglII and BglII/ISceI-tel/BglII (Table S2) were ligated to *Bgl*II-digested pCES1 generating a plasmid (pLS200) with the I-SceI site flanked by telomere seeding sequences in opposite orientations. The structures of the plasmids were confirmed by DNA sequencing. To construct the *ARS^−^* CFV, pADW17 (CFV2/D8B-tg) was cut with *Xma*I and sequences were degraded from the cut site using *E. coli Exo*III to remove the *ARS* and *SUP11* sequences. The digested plasmids were recircularized by ligation and sequenced to determine the extent of resection. One of the resulting plasmids, pSL192, was tested for ability of the *Sna*BI cut, but not uncut, to transform yeast to Ura^+^ prototrophy and the products shown to be exclusively CFs. pRS316:*ADE2* was made by cloning the *Kpn*I/*Sac*1 fragment from pKH5 [51] into the pRS316 vector. The 1.8 kb *Sal*I/*Bgl*II fragment from pLS189, containing the I-SceI cut site inserted at the *Aat*II site [52], was used to replace the *Sal*I/*Bgl*II fragment of pRS316:*ADE2*, generating pLS199. The plasmids pSM502 (*EXO1*) and pSM638 (*exo1-D173A*) were described previously [29].

### BIR assay and telomere seeding assay

Plasmids pCES1 or pLS200 were used to transform wild type or mutant strains, selecting for Ura^+^. Five mL SC-URA glucose cultures of Ura^+^ transformants were grown overnight at 30°C. Cells were diluted to a concentration of 1×10^5^ cells/ml in 350 mL supplemented SD/raffinose medium. Cultures were grown overnight to a concentration of 3×10^6^ cells/mL and galactose was added to the cultures for a final concentration of 2%. Fifty mL of cells were harvested at each indicated time point after galactose induction, DNA was isolated from each sample and digested with *Spe*I or *Pst*I. DNA fragments were separated by electrophoresis through 0.8% agarose gels, transferred to nylon membranes and hybridized with a radiolabeled probe. Cutting efficiency and BIR was assayed with a probe generated by PCR from Chromosome III sequence (coordinates 100791*–*102072) with primers D8BF and D8BR (Table S2). A probe specific for the vector sequences adjacent to the telomere seeding sequence was generated by PCR from primers pADW17F and pADW17R (Table S2) and used for the telomere seeding assays. Samples of cells from each time point were serially diluted and plated on solid SC and SC-URA medium to determine plasmid retention. Because some Ura^+^ colonies are due to uncut plasmids or NHEJ resulting in an unstable plasmid, Ura^+^ colonies were struck onto non-selective YPD medium, then replica plated to SC-URA to determine the stability of the Ura^+^ phenotype. Stable clones were analyzed by PFGE to confirm the presence of the CF. The percent BIR was determined by the number of stable Ura^+^ colonies divided by the number of Ura^+^ colonies normalized to the percent Ura^+^ at 0 hr. The numbers presented in Table 1 are from at least three trials for each strain; significance was determined using an unpaired *t* test

Strand invasion from the CFV was detected by PCR of genomic DNA from cells collected at different times during the induction. PCR was performed with primers CFF2 and CFR3 (Table S2) with Turbo Pfu (Stratagene) and the reaction profile of 92°C for 45 seconds, 55°C for 15 seconds, 68°C for 8 minutes for 25 cycles. The control PCR utilized primers pADW17F and pADW17R.

To detect completed CF events in the time course, 350 ml cultures were grown as above and 50 mL aliquots were collected at each designated time point. Cells were harvested and then prepared for PFGE. These gels were transferred to nylon membrane and hybridized with radiolabled probe generated from D8BF and D8BR as above (Table S2).

To analyze independent repair events cells were grown in SC-URA, transferred to SD/raffinose as above, and then subject to a short (2 h) liquid induction of I-SceI before plating onto medium containing 2% raffinose and 1% galactose with appropriate selection (-URA), or plated directly onto this medium. Individual colonies that arose were tested for stable repair products as above and then prepared for PFGE. These gels were transferred to nylon membranes and hybridized with a radiolabeled probe generated from D8BF and D8BR as above. Statistical significance for aberrant sized CFs was performed using Fisher's exact test.

### Transformation assay for BIR

The chromosome fragmentation vector, pLS192, containing a 5.2-kb insert from the left arm of chromosome III (SGD coordinates 96821*–*102096), was digested with *Sna*BI and 100 ng used to transform competent yeast cells, selecting for Ura^+^ transformants. The frequency of BIR presented in Table 2 is the number of Ura^+^ transformants per microgram of linearized DNA transformed divided by the number of Ura^+^ transformants per microgram of circular pRS416 transformed. The mean BIR frequencies (with standard deviations) presented are from at least 3 independent transformations of each strain.

### DSB–induced gene conversion assay

Five mL SC-URA glucose cultures were grown overnight at 30°C. Cells were diluted to a concentration of 1×10^5^ cells/ml in 350 ml supplemented SD raffinose medium. Cultures were grown overnight to a concentration of 3×10^6^ cells/mL and galactose was added to the cultures for a final concentration of 2%. Fifty mL of cells were harvested at each indicated time point after galactose induction. Samples of cells from each time point were serially diluted and plated on solid SC and SC-URA medium to determine plasmid retention. The percent *URA3* (plasmid) retention was determined by the fraction of Ura^+^ colonies 8 hours after induction normalized to the fraction of Ura^+^ colonies at initiation of the induction. DNA was isolated from each time point and used as template for PCR with primers 3GCA and 5GCA (Table S2) and GoTaq Flexi polymerase (Promega) and the reaction profile of 95°C for 1 minute, 55°C for 1 minute, 72°C for 2 minutes and 30 seconds for 35 cycles. A portion of the PCR reaction was then digested with *Aat*II and analyzed on a 1% agarose gel.

Strand invasion from pLS199 was detected by PCR of genomic DNA from cells collected at different times during the induction. PCR was performed with primers pRS416-2147 and Ade2-2485 (Table S2) with Phusion polymerase (New England Biolabs) and a reaction profile of 98°C for 10 seconds, 55°C for 20 seconds, 72°C for 1 minute 15 seconds for 25 cycles. Reaction products were run on a 1% agarose gel.

## Supporting Information

Table S1 Yeast strains.
(0.04 MB DOC)

Table S2 Oligonucleotides used for plasmid constructions and probes.
(0.09 MB DOCX)
