# Supplementary material for: Extensive DNA End Processing by Exo1 and Sgs1 Inhibits Break-Induced Replication
Source: PLoS Genet. 2010 Jul 8;6(7):e1001007. doi: 10.1371/journal.pgen.1001007 (PMC2900301; doi:10.1371/journal.pgen.1001007)
Supplement: Table S1 — Yeast strains. (0.04 MB DOC) [file pgen.1001007.s001.doc]

Table S1: Yeast strains

| Strain number | Genotype | Source |
| --- | --- | --- |
| LSY1598 | *MAT***a** *lys2::P_GAL_-ISCEI* | S. Marcand |
| LSY2050-21A | *MATα exo1::HIS3 lys2::P_GAL_-ISCEI* | This study |
| LSY2080-59C | *MAT***a** *sgs1::HphMX4 lys2::P_GAL_-ISCEI* | This study |
| LSY2046-34D | *MAT***a** *sgs1::HphMX4 exo1::HIS3 lys2::P_GAL_-ISCEI* | This study |
| LSY2047-29C | *MAT***a** *sgs1::HphMX4 exo1::HIS3 rad51::LEU2 lys2::P_GAL_-ISCEI* | This study |
| LSY2049-12A | *MATα rad51::LEU2 lys2::P_GAL_-ISCEI* | This study |
| LSY2201-12A | *MATα sgs1D664D lys2::P_GAL_-ISCEI* | This study |
| LSY2201-3A | *MATα sgs1D664D exo1::HIS3 lys2::P_GAL_-ISCEI* | This study |
