# Supplementary material for: Extensive DNA End Processing by Exo1 and Sgs1 Inhibits Break-Induced Replication
Source: PLoS Genet. 2010 Jul 8;6(7):e1001007. doi: 10.1371/journal.pgen.1001007 (PMC2900301; doi:10.1371/journal.pgen.1001007)
Supplement: Table S2 — Oligonucleotides used for plasmid constructions and probes. (0.09 MB DOCX) [file pgen.1001007.s002.docx]

Table S2: Oligonucleotides used for plasmid constructions and probes

| Oligo name: | Oligo sequence: |
| --- | --- |
| pCES1-2F | 5’-GATCTTAGGGATAACAGGGTAATCCCACACACCCACACACCCA CACACACACCCACACACCACACAA-3’ |
| pCES1-2R | 5’-AGCTTTGAGTGGTGTGTGGGTGTGTGTGTGGGTGTGTGGGTGT  GTGGGATTACCCTGTTATCCCTAA-3’ |
| BglII/Tel-ISceI/BglII | 5’-GATCTTGTGTGGGTGTGGGTGTGGGTGTGTGGGTGTGTGGGTG  TGTGGATGTGGGTGTGGTGTGGGTGTGGTGTGGGTGTGGTGGA  TTACCCTGTTATCCCTAA-3’ |
| BglII/ISceI-tel/BglII | 5’-GATCTTAGGGATAACAGGGTAATCCACCACACCCACACCACACC  CACACCACACCCACATCCACACACCCACACACCCACACACCCACA  CCCACACCCACACAA-3’ |
| CFF2 (a) | 5’-TATGACCCCCCTTATGCTGG-3’ |
| CFR3 (b) | 5’-TAGGAAGCAGCCCAGTAGTAGG-3’ |
| pADW17F | 5’-CTTATCGATGATAAGCTGTCAAAC-3’ |
| pADW17R | 5’-GCAGCACTGCATAATTCTCTTAC-3’ |
| Ade2-2485 (f) | 5’-CATGGAAAGTACAACAAAATACCGAAACGCC-3’ |
| pRS416-2147R (e) | 5’-CGCGCAATTAACCCTCACTA-3’ |
| D8BF (c) | 5’-CCTATCCCAACTTATTTGCC-3’ |
| D8BR (d) | 5’-GGTTCGGTTGGTGCTTAGG-3’ |
| 3GCA (h) | 5’-CGCCATACTGGAGGCAATAA-3’ |
| 5GCA (g) | 5’-GTTGTGTGGAATTGTGAGCG-3’ |
